# Evaluation of Current Evidence on the Efficacy of Esketamine in Treating Substance-Use Disorders in Patients With Treatment-Resistant Depression (TRD): A Narrative Review

**DOI:** 10.7759/cureus.105219

**Published:** 2026-03-14

**Authors:** Suneha Shelke, Rusheeth R Thummalapally

**Affiliations:** 1 Inflammation and Immunity, Cleveland Clinic Lerner Research Institute, Cleveland, USA; 2 Psychiatry, Mercy Health — BHI St. Rita's Psychiatry, Lima, USA

**Keywords:** esketamine nasal spray, gamma-aminobutyric acid (gaba), n-methyl-d-aspartate, substance use disorder (sud), treatment-related depression

## Abstract

Treatment-resistant depression (TRD) presents a complex clinical challenge, particularly when comorbid with substance use disorders (SUDs) or other compulsive behaviors. With up to a third of major depressive disorder (MDD) patients failing to respond to standard antidepressant therapies, there is growing interest in interventions such as esketamine, a glutamatergic N-methyl-D-aspartate (NMDA) receptor antagonist approved for TRD in 2019, as an intranasal therapy. While esketamine has demonstrated efficacy in alleviating depressive symptoms of TRD, emerging data also point towards its potential in addressing compulsive and addictive behaviors, particularly in patients whose depression and SUDs are deeply intertwined. This review aims to evaluate and synthesize the current literature on the use of esketamine in treating not only TRD, but specifically its application in patients suffering from comorbid substance use and addiction-related behaviors. We aim to clarify the therapeutic mechanisms, examine both human and animal data, and identify whether esketamine offers a dual-modality treatment approach that concurrently reduces depressive symptoms and addictive tendencies. Across peer-reviewed studies, including randomized control trials, cohort analyses, systematic reviews, and preclinical investigations, findings suggest that esketamine may reduce drug-seeking behavior, attenuate cravings, and improve outcomes when combined with behavioral interventions (such as mindfulness-based therapy). In rodent models, esketamine significantly inhibited cocaine-seeking after various abstinence conditions, and clinical data point to its potential role in treating alcohol misuse. In conclusion, esketamine holds potential as a dual-action therapeutic in patients with TRD and comorbid addiction; however, further large-scale studies are needed to explore its therapeutic magnitude, duration of benefit, safety, and effects on substance use-related outcomes.

## Introduction and background

Treatment-resistant depression (TRD), defined as the failure to respond to at least two adequate antidepressant trials, affects up to 30% of major depressive disorder (MDD) patients and is linked to severe functional impairment and increased suicidality [1]. Among these individuals, comorbid substance use disorders (SUDs) and compulsive behaviors present a formidable challenge, often exacerbating treatment resistance and limiting pharmacological options. Esketamine, the S-enantiomer of ketamine and a glutamatergic N-methyl-D-aspartate (NMDA) receptor antagonist, emerged as a promising intervention for TRD and was approved in 2019 by the FDA as an intranasal therapy [2]. Esketamine is thought to have greater NMDA receptor affinity and potentially fewer psychotomimetic effects than ketamine. Esketamine is commercially available under the brand name Spravato® and is administered under medical supervision due to its dissociative effects and potential for misuse. Its use is subject to the FDA’s Risk Evaluation and Mitigation Strategy (REMS) program, which requires administration in certified healthcare settings and post-dose monitoring to mitigate risks of sedation, dissociation, and abuse.

Esketamine offers rapid antidepressant effects and has gained increased attention for its potential to modulate maladaptive reward pathways implicated in addictive and compulsive behaviors. Esketamine shows potential for treating various comorbid conditions, including substance use disorders (SUDs), obsessive-compulsive disorder (OCD), and eating disorders (ED), which frequently co-occur with TRD [3]. The therapeutic mechanisms of esketamine likely involve modulation of glutamatergic neurotransmission and neuroplasticity, which may also underlie its efficacy in reducing addictive behaviors [4]. Early preclinical studies and limited clinical evidence suggest that esketamine may attenuate cravings, reduce compulsive drug-seeking behaviors, and facilitate neuroplastic changes that support recovery from addiction. However, the current literature is fragmented and lacks consensus on its efficacy and safety in this context, especially within TRD populations where neurobiological vulnerability may differ.

**Figure 1:**
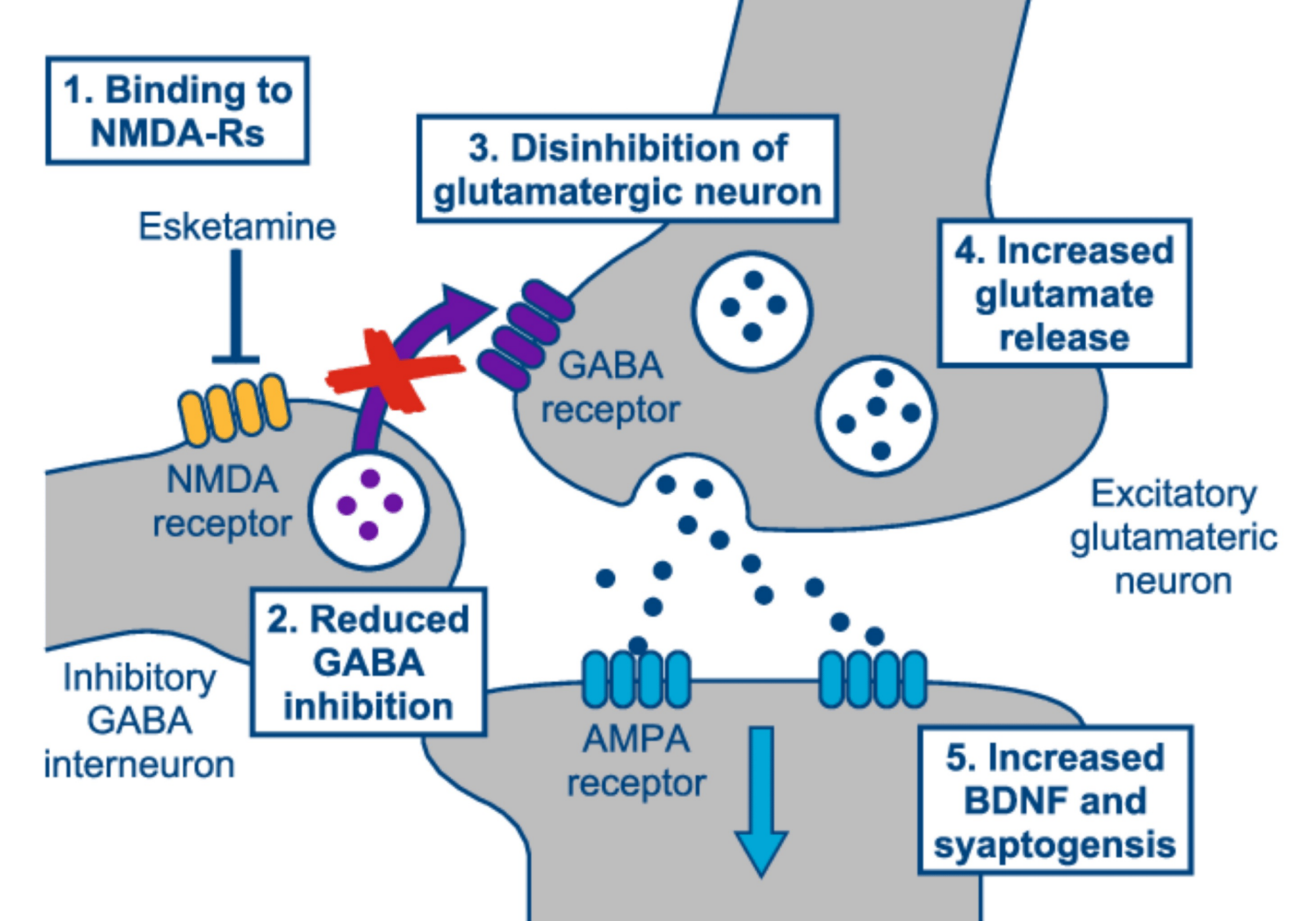
Mechanism of action of esketamine nasal spray AMPA: α-amino-3-hydroxy-5-methyl-4-isoxazolepropionic acid; BDNF: brain-derived neurotrophic factor; GABA: γ-aminobutyric acid, NMDA-R: N-methyl-D-aspartate receptor Adapted from Duman R.S., 2018 [5].

Esketamine nasal spray consists of the S-enantiomer of racemic ketamine, a glutamate receptor antagonist that selectively blocks NMDA receptors expressed on GABAergic inhibitory interneurons, leading to enhanced glutamatergic firing. This, in turn, stimulates AMPA receptor-mediated neurotrophic signaling, which may restore synaptic function in areas of the brain that control mood and emotional behavior (see Figure 1).

Preclinical studies have demonstrated that esketamine can inhibit cocaine-seeking behavior in rats following various abstinence conditions, which is likely mediated by its effects on the glutamatergic system, a critical player in addiction and relapse [6]. Esketamine (at the lower dose of 5 mg/kg more so than 10 mg/kg) reduced cocaine-seeking behavior regardless of whether the rats were kept in social isolation, trained to stop cocaine use (extinction), or in an enriched environment. Cocaine use is known to dysregulate glutamate and dopamine neurotransmission, contributing to the development and persistence of cocaine use disorder (CUD). The article suggests that esketamine may help restore neurochemical balance by modulating glutamatergic signaling.

Key brain regions implicated in relapse and drug-seeking behavior (such as the prefrontal cortex, nucleus accumbens, and hippocampus) undergo withdrawal-associated changes in NMDA receptor subunit composition, particularly involving the GluN2B subunit. Esketamine’s action on these receptors may underlie its observed anti-relapse effects. Additionally, transcriptomic analyses have identified differential gene expression in glutamate-related pathways across various abstinence conditions, further supporting the central role of glutamatergic dysregulation in CUD and its potential as a therapeutic target. The study suggests that esketamine’s ability to modulate glutamate transmission may contribute to its potential as a treatment for SUD in humans [6]. Similarly, a randomized clinical trial combining a single ketamine infusion with mindfulness-based behavioral modification demonstrated efficacy in treating cocaine dependence, with significant reductions in cravings and relapse rates [7].

Mindfulness-based relapse prevention (MBRP) has independently shown efficacy in supporting recovery from SUDs [8]. Combining esketamine with MBRP may provide synergistic benefits, as evidenced in a recent double-blind study investigating the combination of oral esketamine with a mindfulness-based intervention (MBI) in 28 individuals with alcohol use problems [9]. The study aimed to assess outcomes including mindfulness, engagement in MBI, alcohol cravings, and consumption. The results indicated that esketamine significantly enhanced psychological engagement with the daily MBI and led to transient reductions in alcohol cravings, with a significant decrease observed on day 8. However, these effects did not persist. Participants in the esketamine group also reported significantly more mystical experiences and dissociative states compared to those in the placebo group. However, there were no significant differences between the groups in terms of alcohol consumption or self-reported mindfulness practice.

While the transient effects on alcohol cravings are promising, further research is needed to evaluate long-term efficacy. The study provides preliminary evidence supporting the potential benefits of integrating esketamine with mindfulness-based interventions, but larger sample sizes and longer follow-up periods are essential to confirm these effects and explore the underlying mechanisms [9]. Supporting this, another study evaluated adjunctive ketamine treatment alongside relapse prevention psychological therapy in alcohol use disorder, finding significant improvements in abstinence rates and reductions in cravings [10].

Recently analysis of the data from the FDA Adverse Event Reporting System (FAERS) was done to explore potential associations between ketamine, esketamine, and substance use or alcohol-related adverse events [11]. While both agents are approved for TRD, concerns persist regarding their abuse liability. In this analysis, ketamine was associated with significantly elevated reporting odds ratios (RORs) for several adverse outcomes, including substance dependence (ROR = 18.72), substance use disorder (ROR = 11.40), and drug abuse (ROR = 3.72). These findings reinforce existing concerns about ketamine’s potential for misuse. Yet another report further extended this safety assessment by comparing adverse effects between genders using FAERS data, revealing that females reported higher rates of dizziness, nausea, and dissociation, while males exhibited more frequent reports of increased blood pressure and tachycardia [12].

Earlier research on ketamine psychedelic therapy (KPT) [13, 14] provided foundational evidence for ketamine’s therapeutic potential in addiction by facilitating psychotherapeutic engagement and disrupting addictive patterns [13]. Additional complementary interventions, such as meditative movement and mindfulness practices, have been studied for depression and anxiety management, potentially augmenting esketamine’s benefits [15]. Collectively, these studies underscore esketamine’s multifaceted role as both a rapid antidepressant and a promising adjunct in treating SUDs, particularly when integrated with behavioral therapies like mindfulness-based relapse prevention.

Conversely, esketamine demonstrated significantly reduced RORs for substance abuse (ROR = 0.37), drug dependence (ROR = 0.13), and drug abuse (ROR = 0.048), suggesting a potentially lower post-marketing misuse signal and a more favorable risk profile [11]. The authors note, however, that FAERS data cannot establish causality and may be influenced by reporting biases and market exposure differences. The researchers suggest that both drugs benefit substance misuse by affecting brain systems involved in reward function, cognition, and impulse control.

The current evidence base suggests that esketamine holds meaningful promise as a dual-action therapeutic in patients with TRD and comorbid SUD. Its rapid-acting antidepressant properties, coupled with emerging data on its effects on craving and relapse-related neurobiology, point to a potentially transformative role in this complex patient population.

Preclinical studies demonstrate that esketamine attenuates relapse-like drug-seeking behavior, preventing morphine preference reacquisition and inhibiting cocaine-seeking across multiple abstinence conditions in rat models, thereby supporting its potential role in reducing vulnerability to substance use relapse [6, 16]. Clinical trials support esketamine’s capacity to enhance engagement with psychotherapeutic interventions, such as mindfulness-based therapy, possibly by increasing emotional openness and psychological insight through transient dissociative and mystical experiences. These findings parallel those from psychedelic-assisted therapy models. Despite these encouraging results, several limitations persist, including small sample sizes and variable protocols. The transient nature of esketamine’s effect on cravings underscores the importance of longitudinal research to determine sustained benefits. Moreover, while post-marketing data suggest a lower misuse liability for esketamine compared to ketamine, these findings must be interpreted cautiously due to the passive, voluntary nature of FAERS reporting.

## Review

Methods

We conducted a comprehensive review of the available literature examining the use of esketamine in the management of treatment-resistant depression (TRD) and comorbid substance use disorders. Studies were included if they met the following criteria: (1) focused on the use of esketamine or ketamine in treating substance-use disorders (SUDs), particularly in patients diagnosed with treatment-resistant depression (TRD); (2) reported on efficacy outcomes, such as reduction in substance use, craving intensity, or depression severity; and (3) were original research articles (e.g., randomized controlled trials, cohort studies, or case series). Exclusion criteria were not pre-specified, but studies were screened for relevance and excluded if they clearly did not meet inclusion criteria, such as those focused solely on anesthesia or unrelated psychiatric conditions. Excluded were also studies not involving TRD populations or not reporting outcomes relevant to substance use, cravings, or addiction-related behaviors. Case reports, editorials, and conference abstracts without full data were excluded. A comprehensive literature search was conducted in May 2025 across three major electronic databases: PubMed, Google Scholar, and the National Institutes of Health (NIH) Research Portfolio Online Reporting Tools (RePORTER). No date restrictions were applied to capture both early and recent developments in the field. Secondary manual searches of reference lists from all included studies were performed to identify additional relevant articles. The search strategy combined keywords and Boolean operators tailored to each database. Keywords included: “treatment-resistant depression” OR “TRD,” “substance use disorder” OR “substance abuse,” “esketamine,” “ketamine,” “alcohol use,” and “cravings.” A reviewer screened titles and abstracts for relevance, followed by a full-text review of potentially eligible studies.

Data were extracted using a standardized form; extracted information included study design, sample characteristics, intervention details (dose, frequency, and duration of esketamine/ketamine treatment), comparator conditions (if applicable), outcome measures related to SUDs and depression, and primary findings. The primary outcomes of interest were changes in substance use behavior, craving intensity, and addiction-related symptoms following esketamine treatment in individuals with TRD. Secondary outcomes included engagement in behavioral therapies, mystical/dissociative experiences, and adverse event profiles, particularly substance misuse or dependency related to esketamine. Given the heterogeneity in study designs, formal risk of bias assessment was not applied systematically, but potential biases (e.g., small sample size, lack of control groups) were noted and considered in the narrative synthesis. Due to variability in study design, populations, and outcome measures, a narrative synthesis approach was employed. Findings were synthesized qualitatively, organized thematically by (1) preclinical findings, (2) clinical outcomes in TRD with comorbid SUD, and (3) post-marketing pharmacovigilance data. The narrative synthesis considered both convergent and divergent findings across studies and discussed potential mechanisms of action and clinical implications.

Results and discussion

This review identified 10 studies meeting inclusion criteria, comprising two preclinical (animal) studies, five randomized controlled trials (RCTs), and three observational/cohort studies. Sample sizes ranged from 12 to 350 participants, with most studies enrolling adults aged 18-65 diagnosed with TRD and varying rates of comorbid SUD. Interventions typically involved intranasal esketamine administered at doses of 28 mg, 56 mg, or 84 mg twice weekly initially, followed by maintenance dosing. Control conditions included placebo nasal sprays or oral antidepressants alone. These studies investigated esketamine’s potential therapeutic role in addressing both treatment-resistant depression (TRD) and substance use disorders (SUDs), either as a primary or secondary focus. Preclinical studies consistently demonstrated esketamine’s efficacy in modulating neurobiological pathways implicated in addictive behavior. In rodent models, esketamine significantly reduced cocaine-seeking behavior across various abstinence conditions (including social isolation, extinction training, and enriched environments), suggesting effects may generalize across various abstinence conditions. Authors reported that esketamine at lower doses (5 mg/kg) more effectively inhibited relapse behaviors compared to higher doses, indicating a possible dose-dependent effect mediated through NMDA receptor subunit modulation, particularly GluN2B [6]. Transcriptomic analysis in these models identified altered glutamatergic signaling, suggesting neuroplastic adaptations that support reduced drug-seeking behaviors.

Several clinical studies highlighted esketamine’s ability to influence substance-related behaviors in patients with TRD. A recent randomized, double-blind study looked at esketamine combined with mindfulness-based intervention (MBI) in individuals with problematic alcohol use. Esketamine significantly enhanced psychological engagement in MBI sessions and produced transient reductions in alcohol craving, peaking around day 8 post-administration [9]. However, changes in alcohol consumption levels and mindfulness practice adherence were not statistically significant, and the effect on cravings diminished by the study’s end. A randomized, double-blind clinical trial explored the dose-dependent effects of esketamine in patient-controlled analgesia after major lumbar fusion surgery, finding that esketamine reduced opioid consumption in a dose-dependent manner [17]. Other observational studies and open-label trials suggested that esketamine may reduce opioid and stimulant use when administered in structured clinical environments. These effects were often augmented when combined with psychotherapy or other behavioral interventions, suggesting a synergistic treatment paradigm. Notably, there is limited but growing evidence of esketamine’s potential to facilitate treatment retention and reduce dropout rates in dual-diagnosis populations (TRD + SUD), though most of these findings remain preliminary and underpowered.

Analysis of post-Marketing and Pharmacovigilance Data from the FDA Adverse Event Reporting System (FAERS) showed a markedly different risk profile between ketamine and esketamine [11]. While ketamine was associated with elevated reporting odds ratios (RORs) for substance use disorder (11.40), drug dependence (18.72), and abuse (3.72), esketamine exhibited substantially lower RORs for similar adverse outcomes: substance abuse (0.37), dependence (0.13), and abuse (0.048). These findings suggest that esketamine, when administered in a regulated, medically supervised setting, may carry a comparatively lower risk for misuse or diversion. The favorable post-marketing risk profile of esketamine, as reflected in FAERS data, may be partly attributable to safeguards imposed by the REMS program, which ensures its use occurs under controlled conditions, including certified healthcare settings and post-dose observation. However, pharmacovigilance studies are inherently limited by underreporting, selection biases, and confounding due to differential drug access and population exposure. Hence, while the data are promising, they are not definitive.

The current evidence base suggests that esketamine holds meaningful promise as a dual-action therapeutic in patients with TRD and comorbid SUD. Its rapid-acting antidepressant properties, coupled with emerging data on its effects on craving and relapse-related neurobiology, point to a potentially transformative role in this complex patient population.

The mechanism by which esketamine may exert anti-addictive effects likely involves the modulation of glutamatergic signaling pathways, particularly in the prefrontal cortex, nucleus accumbens, and hippocampus - regions integrally involved in executive function, reward processing, and relapse. Unlike traditional antidepressants that primarily act on monoaminergic systems, esketamine’s NMDA receptor antagonism and downstream activation of AMPA receptors may promote synaptic plasticity and cognitive flexibility, which are crucial for addiction recovery.

Clinical trials support esketamine’s capacity to enhance engagement with psychotherapeutic interventions, such as mindfulness-based therapy, possibly by increasing emotional openness and psychological insight through transient dissociative and mystical experiences. These paralleled findings from psychedelic-assisted therapy models, where altered states of consciousness are posited to catalyze therapeutic change. Despite these encouraging findings, several limitations persist. Clinical studies are few, often small in sample size, and highly variable in terms of dosing protocols, outcome measures, and follow-up duration. The transient nature of esketamine’s effect on cravings underscores the importance of longitudinal research to determine sustained benefits [9]. Moreover, while post-marketing data suggest a lower misuse liability for esketamine compared to ketamine, these findings must be interpreted with caution due to the passive, voluntary nature of FAERS reporting. The heterogeneous definitions of TRD and SUD across studies also pose a challenge for synthesizing findings. Future research should prioritize standardized diagnostic criteria, explore personalized treatment algorithms (e.g., based on genetic or neuroimaging biomarkers), and evaluate long-term safety and efficacy in real-world settings. The key findings of the trial discussed in this review are summarized in Table 1.

**Table 1:** Summary of Study Results TRD: Treatment-resistant depression; RCT: Randomized controlled trial; MBI: Mindfulness-based intervention; SUD: Substance use disorder; FAERS: FDA Adverse Event Reporting System

Study	Type	Population / Model	Intervention	Findings / Outcome
Wydra et al., 2023 [6]	Preclinical	TRD + opioid/stimulant use	Esketamine 5 mg/kg vs 10 mg/kg	Reduced cocaine-seeking; 5 mg/kg more effective
Fontoura et al., 2025 [16]	Animal	Wistar rats	Esketamine	Reduced the reacquisition of morphine preference indicating a protective effect against relapse-like behavior
Dakwar et al., 2019 [7]	RCT	Cocaine use disorder	Ketamine + mindfulness	Reduced cravings and relapse
Gent et al., 2024 [9]	RCT	Alcohol use disorder + TRD	Oral Esketamine + MBI	Increased MBI engagement, transient craving reduction; more mystical/dissociative experiences
Grabski et al., 2022 [10]	RCT	Alcohol use disorder	Ketamine + relapse prevention therapy	Increased abstinence, reduced cravings
Brinck et al., 2021 [17]	RCT	Post-operative patients	Esketamine (PCA)	Dose-dependent opioid reduction
Daly et al., 2019 [2]	RCT	Adults with TRD ± SUD	Esketamine vs placebo/antidepressants	Positive outcomes on TRD; mixed for SUD
Gutierrez et al., 2025 [18]	Observational	TRD + SUD	Esketamine	Low risk of abuse or misuse
Yang & Chen, 2024 [12]	FAERS analysis	Gender subgroups	Esketamine AE	Women: dizziness/nausea; Men: hypertension/tachycardia
Krupitsky et al., 2007 [14]	Open-label	SUD	Ketamine-assisted psychotherapy	Improved engagement; reduced relapse

## Conclusions

Esketamine shows promise as a treatment for patients with treatment-resistant depression and comorbid substance use disorders. Evidence suggests it may reduce cravings and drug-seeking behavior, potentially through modulation of glutamatergic neurotransmission and enhancement of neuroplasticity. Combining esketamine with behavioral interventions like mindfulness therapy may further support recovery. While preliminary findings are encouraging, larger-scale clinical trials are needed to confirm long-term efficacy and ensure safety. Overall, esketamine may be a valuable component of integrated treatment strategies, particularly for populations with few other effective options.
